# The Tibetan Medicinal Formula Ruyi Zhenbao: Research progress on pharmacological actions, clinical evidence and compatibility

**DOI:** 10.3389/fphar.2026.1768770

**Published:** 2026-09-02

**Authors:** Cairang Xiadao, Pengcuo Danzhi, Tsedien Nhamdriel, Dongzhi Yangchang, Chenlei Ganghuan, Yongzhong Zeweng, Zhang Wang, Ma Mi

**Affiliations:** 1 School of Ethnic Medicine, Chengdu University of Traditional Chinese Medicine, Chengdu, China; 2 Department of Tibetan Medicine, Tibet University of Tibetan Medicine, Lhasa, China

**Keywords:** clinical application, formula compatibility analysis, pharmacological action, Ruyi Zhenbao formula, Tibetan medicine, white channel disorder

## Abstract

**Objective:**

To comprehensively review the research progress on the compatibility principles, pharmacological mechanisms, and clinical evidence of the Tibetan medicinal formula Ruyi Zhenbao.

**Methods:**

Guided by fundamental Tibetan medical theory, a critical review of domestic and international literature was conducted, systematically integrating Tibetan medicinal compatibility theory, preclinical pharmacological evidence, and clinical research data.

**Results:**

Based on the multi-level medicinal property compatibility structure of Tibetan medicine, this formula combines 30 medicinal materials according to their properties and effects, forming a synergistic therapeutic system with heat-clearing, mind-awakening, collateral-dredging, and serous-fluid-drying properties. Preclinical studies indicate that Ruyi Zhenbao Formula may exert integrative regulatory effects through multiple targets and pathways, demonstrating multiple pharmacological activities including neuroprotection and repair, anti-inflammatory and immunomodulation, multi-mechanism synergistic analgesia, and microcirculation improvement. Clinical evidence suggests that this formula demonstrates potential efficacy in conditions such as osteoarthritis, cerebrovascular diseases, and neurological disorders, with acceptable safety profiles based on available data.

**Conclusion:**

Current preclinical and clinical evidence supports the traditional applications of the Ruyi Zhenbao Formula and provides preliminary scientific insights into the Tibetan medical theories underlying its use. This review contributes to understanding the potential of integrating traditional Tibetan medical knowledge with modern biomedical research. However, a ConPhyMP-based quality assessment showed that none of the included primary studies reported voucher specimens or chemical fingerprints, underscoring the need for standardized phytochemical characterization in future research.

## 1 Introduction

Ruyi Zhenbao Formula (pill, tablet, abbreviated as RYZBF, བསམ་འཕེལ་ནོར་བུ) is a classic and renowned Tibetan medicinal formula for treating “White Channel Disorder.” (རྩ་དཀར་ནད) Its formula lineage can be traced back to the “Twenty-Five Ingredient Pearl Powder” (ཉ་ཕྱིས་ཉེར་ལྔ) recorded in the eighth-century Tibetan medical classic “*The Four Tantras*” (རྒྱུད་བཞི), representing over a thousand years of evolutionary application. In the 17th century, Dammam Ramba Lobsang Chödrak (དར་མོ་སྨན་རམས་པ་བློ་བཟང་ཆོས་གྲགས) and his disciples developed the basic formula and first named it “bsam ‘phel nor bu” (Ruyi Zhenbao). In the 19th century, Chenrezig Norbu (མཁྱེན་རབ་ནོར་བུ) further supplemented and refined it, forming the 30-ingredient RYZBF that has been used ever since (An et al., 2025), In 1995, it was officially included in the *Drug Standard of the Ministry of Health of the People’s Republic of China (Tibetan Medicine Volume)*, establishing it as a legally recognized standard formula. Composed of 30 medicinal substances including *Lagotis brachystachya*, *Margaritifera*, *Crocus sativus*, *Myristica fragrans*, *Terminalia bellirica*, *Piper longum*, *Cinnamomum cassia*, *Inula racemosa*, artificial *Moschus*, *Aquilaria sinensis*, *Terminalia chebula*, and *Cassia obtusifolia*, this formula is indicated for clearing heat, awakening the mind and opening the orifices, relaxing sinews and dredging collaterals, and drying serous fluid. “White Channel Disorder” (also known as Bai Mai disease) is a core concept in Tibetan medicine, referring to channels originating from the brain with rLung as their material basis, which from a modern medical perspective broadly correspond to the central and peripheral nervous systems. Consequently, therapies targeting White Channel Disorder hold significant value in treating neurological and osteoarticular diseases (Chen et al., 2024).

The modernization of Tibetan medicinal formulas is not only essential for the preservation and development of ethnic medicine but also offers a unique therapeutic paradigm of “multi-component, multi-target, holistic regulation” for addressing complex, multi-factorial diseases. Recent years have witnessed substantial progress in RYZBF research: pharmacological studies have confirmed its neuroprotective, anti-inflammatory, analgesic, and microcirculation-improving activities, while clinical investigations have demonstrated its efficacy in osteoarthritis, cerebrovascular diseases, and neuropathic pain. Concurrently, the scientific basis of Tibetan medical theories such as “Taste, Property, and Post-Digestive Taste” (རོ་ནུས་ཞུ་རྗེས) and formula compatibility is gradually being elucidated—the synergistic action of heat-clearing, mind-awakening, collateral-dredging, and serous-fluid-drying (ཆུ་སེར་སྐེམ་པ) agents within the formula creates a multi-target integrative therapeutic profile that aligns closely with the principles of modern network pharmacology (Zhuoma Cuo et al., 2023; Ge et al., 2022; Gong et al., 2019).

However, critical challenges persist: the scientific interpretation of its compatibility principles remains unclear, with insufficient integration of traditional theory and modern mechanisms; research on phytochemical metabolites and pharmacodynamic material basis is still preliminary; and a comprehensive review integrating traditional theory with modern evidence through critical assessment is currently lacking, hindering a full understanding of progress in this field. Therefore, this review critically examines domestic and international literature guided by Tibetan medicinal property compatibility and White Channel Disorder theory, aiming to provide an integrative critical evaluation of RYZBF’s compatibility principles, pharmacological actions, and clinical research from the intersecting perspective of traditional theory and modern science. This article attempts to comprehensively map core Tibetan medical concepts—“awakening the mind and opening the orifices,” “drying serous fluid,” and “relaxing sinews and dredging collaterals”—onto modern pharmacological mechanisms including neuroprotection, anti-inflammation, and analgesia. Furthermore, it employs the ConPhyMP tool to conduct quality assessment and critical analysis of existing evidence, constructing a comprehensive framework encompassing compatibility principles, phytochemistry, pharmacological mechanisms, clinical applications, evidence limitations, and future directions. This work aims to provide a theoretical foundation for further research and rational clinical application of RYZBF, while offering a methodological reference for the study of other ethnic medicine formulas.

## 2 Methods

### 2.1 Literature search strategy

The following databases were systematically searched from their inception to 1 February 2026: PubMed, Web of Science, China National Knowledge Infrastructure (CNKI), Wanfang Data, and VIP Chinese Journal Database. Search terms included Chinese keywords: “如意珍宝,” “如意珍宝丸,” “如意珍宝片,” “桑培诺布,” “藏药,” “白脉病,” and their English counterparts: “Ruyi Zhenbao,” “Ruyi Zhenbao Pill,” “Ruyi Zhenbao Tablet,” “Sangpei Nuobu,” “bsam ‘phel nor bu,” “Tibetan medicine,” “White Channel Disorder.” Additional terms related to pharmacological effects and disease areas—such as “neuroprotection,” “anti-inflammatory,” “analgesia,” “osteoarthritis,” and “cerebrovascular diseases”—were used in combination with Boolean operators (AND, OR) to ensure comprehensive coverage. The reference lists of included articles were also manually searched to supplement relevant studies.

### 2.2 Literature screening and quality assessment

#### 2.2.1 Inclusion criteria

(1) Studies addressing the pharmacological effects, clinical applications, or chemical metabolites of the Ruyi Zhenbao Formula; (2) original research (*in vitro*, animal, or clinical studies) or systematic reviews; (3) published in peer-reviewed journals; (4) written in Chinese or English. Narrative reviews, expert commentaries, opinion articles, conference abstracts, and studies unrelated to the topic were excluded.

#### 2.2.2 Quality assessment

Following the “Four Pillars of Best Practice in Ethnopharmacology” of Frontiers in Pharmacology, the ConPhyMP tool was employed to evaluate the included pharmacological studies. ConPhyMP Table 1 was used to assess the completeness of reporting for species names (including authorities), medicinal parts, voucher specimens, material sources, and extraction processes. Table 2 (applicable to pharmacopoeia-listed medicinal materials) was used to evaluate the provision of chemical fingerprints, quantification of marker compounds, or commercial batch numbers. Detailed assessment results are presented in Supplementary Tables S1,S2, provided in Excel format. All species names were verified against the Kew MPNS database and the Pharmacopoeia of the People’s Republic of China (2020 edition). For taxonomically revised species (e.g., *Amomum kravanh, Amomum tsao-ko*), the official names from the Chinese Pharmacopoeia were retained. A ConPhyMP assessment was accordingly performed on the included primary pharmacological studies; it revealed that none of the included studies reported voucher specimens or chemical fingerprints, with only a limited number quantifying marker compounds (Supplementary Tables S1,S2). These limitations are examined further in the Discussion (Limitations and Future Directions).

**TABLE 1:** List of formula categories, medicinal properties and efficacies of RYZBF.

No.	Formulation category	Formulation ingredient	Latin name (with author)	Family	Part used (pharmaceutical name)	Taste/property	Efficacy
1	Mind-awakening and orifice-opening	Tabasheer	*Bambusa textilis McClure*	Poaceae	Bambusae Concretio Silicea	Sweet, cold	Clears heat, transforms phlegm, calms fright
2		Saffron/Crocus sativus	*Crocus sativus L.*	Iridaceae	Croci Stigma	Sweet, neutral	Activates blood, resolves stasis, cools blood, detoxifies, tranquilizes mind
3		Clove	*Syzygium aromaticum (L.) Merr. and L.M.Perry*	Myrtaceae	Caryophylli Flos	Pungent, warm	Warms the middle, promotes qi circulation, treats life-channel diseases
4		Nutmeg	*Myristica fragrans Houtt.*	Myristicaceae	Myristicae Semen	Pungent, warm	Warms the middle, promotes qi circulation, astringes intestine, treats rLung disorders
5		Liquorice root	*Glycyrrhiza uralensis Fisch.*	Fabaceae	Glycyrrhizae Radix et Rhizoma	Sweet, neutral	Treats lung diseases, benefits the nerves
6		Shortspike lagotis	*Lagotis brachystachya Maxim.*	Plantaginaceae	Lagotis Herba	Sweet, bitter, cool	Clears heat, detoxifies, absorbs serous fluid and pus-blood
7	Heat-clearing	Round cardamom	*Amomum kravanh Pierre ex Gagnep.*	Zingiberaceae	Amomi Fructus Rotundus	Pungent, warm	Transforms dampness, promotes qi circulation, warms the middle, treats kidney diseases
8		Tsao-ko/black cardamom	*Amomum tsao-ko Crevost et Lemaire*	Zingiberaceae	Tsaoko Fructus	Pungent, warm	Dries dampness, warms the middle, interrupts malaria, removes phlegm
9		White sandalwood	*Santalum album L.*	Santalaceae	Santali Albi Lignum	Astringent, pungent, cool	Clears lung fire, treats heart heat, clears heat and detoxifies
10		Red sandalwood	*Pterocarpus santalinus L.f.*	Fabaceae	Pterocarpi Lignum	Astringent, cool	Clears blood heat
11		Agarwood	*Aquilaria sinensis (Lour.) Gilg*	Thymelaeaceae	Aquilariae Lignum Resinatum	Bitter, pungent, cool	Promotes qi circulation, relieves pain, warms the middle, stops vomiting
12		Artificial musk	*Moschus berezovskii Flerov*	Moschidae	Moschus (Artificial)	Pungent, warm	Resuscitation, activates blood, unblocks meridians, reduces swelling
13		Cow bezoar	*Bos taurus domesticus Gmelin*	Bovidae	Calculus Bovis	Sweet, bitter, cool	Clears heat, detoxifies, cools the liver
14		Nacre (pearl shell)	*Margaritifera (L.)*	Margaritiferidae	Concha Margaritifera	Astringent, cool	Benefits the brain, detoxifies, treats White Channel Disorder, calms the mind
15		Buffalo horn	*Bubalus bubalis Linnaeus*	Bovidae	Cornu Bubali	Sweet, cool	Clears heat, cools blood, detoxifies, absorbs serous fluid
16		Cumin	*Cuminum cyminum L.*	Apiaceae	Cymini Fructus	Pungent, sweet, warm	Clears lung heat
17		Glandular-hair nigella	*Nigella glandulifera Freyn and Sint.*	Ranunculaceae	Nigellae Semen	Sweet, pungent, warm	Treats liver cold
18		Inula root	*Inula racemosa Hook.f.*	Asteraceae	Inulae Radix	Sweet, bitter, pungent, neutral	Clears heat, detoxifies, warms stomach, relieves pain
19		Vladimiria root	*Vladimiria souliei (Franch.) Ling*	Asteraceae	Vladimiriae Radix	Bitter, pungent, warm	Regulates abdominal pain, treats rLung-mKhris-pa disorders
20	Middle-warming and three nyépa harmonizing	Chebulic myrobalan	*Terminalia chebula Retz.*	Combretaceae	Chebulae Fructus	Astringent, neutral	Nourishes body, harmonizes Three Nyépa, aids digestion
21		Belleric myrobalan	*Terminalia bellirica (Gaertn.) Roxb.*	Combretaceae	Belliricae Fructus	Astringent, bitter, neutral	Treats Bad-kan-mKhris-pa disorders, absorbs serous fluid
22		Emblic myrobalan	*Phyllanthus emblica L.*	Phyllanthaceae	Phyllanthi Fructus	Sour, astringent, sweet, warm	Treats Bad-kan-mKhris-pa disorders, treats blood diseases
23		Long pepper	*Piper longum L.*	Piperaceae	Piperis Longi Fructus	Pungent, sweet, warm	Treats various cold syndromes
24		Galangal	*Alpinia officinarum Hance*	Zingiberaceae	Alpiniae Officinarum Rhizoma	Pungent, sweet, warm	Generates heat, treats spleen disorders
25		Cinnamon bark	*Cinnamomum cassia (L.) J.Presl*	Lauraceae	Cinnamomi Cortex	Sweet, pungent, warm	Treats stomach cold, liver cold
26	Serous-fluid-drying	Amber	(mineral)	—	Succinum	Sweet, neutral	Detoxifies, absorbs serous fluid
27		Cassia seed	*Cassia obtusifolia L.*	Fabaceae	Cassiae Semen	Bitter, cool	Counteracts serous fluid, treats skin diseases
28		Musk mallow	*Abelmoschus moschatus Medik.*	Malvaceae	Abelmoschi Herba	Pungent, sweet, cool	Clears heat, promotes diuresis, reduces swelling
29		Lygodium spores	*Lygodium japonicum (Thunb.) Sw.*	Lygodiaceae	Lygodii Spora	Sweet, cold	Promotes diuresis, treats kidney diseases
30	Sinew-relaxing and collateral-dredging	Crab	*Eriocheir sinensis H. Milne-Edwards*	Varunidae	Eriocheir	Sweet, salty, warm	Tonifies kidney, promotes diuresis, relaxes sinews

All species names were verified against the Kew MPNS, database and the Pharmacopoeia of the People’s Republic of China (2020 edition). Formulation categories, medicinal properties, and efficacies are primarily based on the Drug Standard of the Ministry of Health of the People’s Republic of China (Tibetan Medicine Volume) (Ministry of Health of the People’s Republic of China, 1995) and relevant Tibetan medical literature (Tenzin Phuntsok, 2005; Zhuoma Cuo et al., 2023).

For taxonomically revised species (e.g., Amomum kravanh, Amomum tsao-ko), the traditional names accepted by the Chinese Pharmacopoeia are retained as they are the legally recognized names in the source standard.

**TABLE 2:** Summary of chemical metabolite studies on RYZBF.

Study	Analytical method	Metabolites/Classes identified	Source materials	Key findings	Limitations	References
Li et al. (2023)	HPLC	Chebulagic acid, ellagic acid, eutannin, quercetin, kaempferol, isoalantolactone (6 metabolites)	*Terminalia chebula*, *Phyllanthus emblica*, *Glycyrrhiza uralensis*, *Inula racemosa*	Quantified ellagic acid (2.02 mg/g) and eutannin (5.25 mg/g)	Only six metabolites analyzed; complete fingerprint not provided; single HPLC method without MS confirmation	Li et al. (2023)
Hou et al. (2024a)	HPLC-MS/MS	Agarotetrol, isoliquiritigenin, piperine, ferulic acid (4 metabolites)	*Aquilaria sinensis*, *Glycyrrhiza uralensis*, *Piper longum*, *Ferula* spp.*	Pharmacokinetic study; confirmed distribution to brain, heart, liver, and kidney tissues	Only four known metabolites tracked; no novel metabolite discovery; complete fingerprint not provided	Hou et al. (2024a)
Chen et al. (2024)	UHPLC-Q-orbitrap HRMS	Flavonoids and their glycosides, alkaloids, terpenoids, organic acids (metabolite classes); 126 metabolites identified *in vitro*	Multiple formula source materials	Systematic *in vitro* metabolite identification; confirmed presence of multiple metabolite classes	*In vitro* analysis only; no quantification; structural confirmation of individual metabolites not performed	Chen et al. (2024)
Xu et al. (2024)	UHPLC-Q-TOF-MS/MS	13 brain-penetrating metabolites (daidzin, cholic acid, piperine, etc.), with 5 verified by reference standards	*Dalbergia odorifera*, *Piper longum*, *Glycyrrhiza uralensis* extract, *Cinnamomum cassia*, *Inula racemosa*, *Abelmoschus moschatus*, *Bos taurus domesticus*, *Crocus sativus*	First identification of brain-penetrating metabolites; 5 metabolites verified with reference standards	No quantitative analysis; source attribution not fully established; no correlation with pharmacological effects	Xu et al. (2024)
Hou et al. (2022)	HPLC-MS/MS	Agarotetrol, isoliquiritigenin, piperine, ferulic acid, and tissue-distributed metabolites (14 in brain, 18 in heart, 24 in liver, 24 in kidney)	Same as formula composition	Systematic study of single- and multiple-dose pharmacokinetics; clarified tissue distribution patterns	Targeted tracking of known metabolites; non-targeted metabolomics analysis not performed	Hou et al. (2022)
Han et al. (2017)	TLC + HPLC	Gallic acid, dehydrodiisoeugenol, piperine, cinnamaldehyde (quantified); TLC identification of 12 medicinal materials	*Terminalia chebula*, *Dalbergia odorifera*, *Inula racemosa*, *Aquilaria sinensis*, *Piper longum*, *Syzygium aromaticum*, *Alpinia officinarum*, *Crocus sativus*, *Cinnamomum cassia*, *Cassia obtusifolia*, *Boswellia* spp., artificial *Bos taurus domesticus*	Established multi-index quality control method; method demonstrated strong specificity and good reproducibility	Targeted only a limited number of marker compounds; failed to fully reflect the overall chemical profile of the formula	Han et al. (2017)

This table summarizes representative studies only; for detailed metabolite listings, please refer to the original publications.

* Source materials for certain metabolites were inferred based on formula composition and literature reports.

### 2.3 Review type

This article is a critical review aimed at comprehensively and critically evaluating the existing evidence on RYZBF, rather than a systematic review with meta-analysis. Its objective is to provide a comprehensive overview of the formula’s compatibility principles, pharmacological mechanisms, and clinical applications, while identifying knowledge gaps and future research directions.

## 3 Compatibility and formula analysis

### 3.1 Formula composition

According to the *Drug Standard of the Ministry of Health of the People’s Republic of China (Tibetan Medicine Volume, First Edition)* (Ministry of Health of the People’s Republic of China, 1995), the composition and dosage of RYZBF are as follows: Tabasheer (ཅུ་གང་) 100 g, Stigma Croci (གུར་གུམ) 100 g, Flos Caryophylli (ལི་ཤུ) 40 g, Semen Myristicae (ཛཱ་ཏི) 40 g, Fructus Amomi (སུག་སྨེལ) 40 g, Fructus Tsaoko (ཀ་ཀོ་ལ) 30 g, Lignum Santali Albi (ཙན་དན་དཀར་པོ) 80 g, Lignum Pterocarpi (ཙན་དན་དམར་པོ) 330 g, Lignum Aquilariae Resinatum (ཨ་གར) 100 g, Moschus (གླ་རྩི) 2 g, Calculus Bovis (གི་ཝང་) 2 g, Concha Margaritifera (ཉ་ཕྱིས) 100 g, Cornu Bubali (མ་ཧེ་རྭ) 40 g, Fructus Chebulae (ཨ་རུ) 30 g, Fructus Belliricae (བ་རུ) 100 g, Fructus Phyllanthi (སྐྱུ་རུ་ར) 130 g, Fructus Cuminum (ཟི་ར་དཀར་པོ) 40 g, Semen Nigellae (ཟི་ར་ནག་པོ) 40 g, Fructus Piperis Longi (པི་པི་ལིང་) 30 g, Rhizoma Alpiniae Officinarum (སྒ་སྨུག) 80 g, Cortex Cinnamomi (ཤིང་ཚ) 50 g, Succinum (སྤོས་དཀར) 60 g, Semen Cassiae (ཐལ་ཀ་རྡོ་རྗེ) 60 g, Herba Abelmoschi (སོ་མ་ར་ཛཱ) 50 g, Radix Inulae (མ་ནུ) 80 g, Radix Vladimiriae (རུ་རྟ) 80 g, Radix Glycyrrhizae Extract (ཤིང་མངར་ཁན་ཌ) 40 g, Herba Lagotis Brachystachyae (འབྲི་ཏ་ས་འཛིན) 150 g, Spora Lygodii (གསེར་བྱེ) 30 g, and Eriocheir (སྡིག་སྲིན) 50 g.

### 3.2 Analysis of compatibility and formula theory

Tibetan medicinal formula compatibility is generally based on the characteristics of “Taste, Property, and Post-Digestive Taste” and efficacy, forming three major principles: Taste Compatibility (རོ་སྡེབ), Post-Digestive Taste Compatibility (ཞུ་རྗེས་སྡེབ་པ), and Property/Efficacy Compatibility (ནུས་སྡེབ). These principles follow a progressive sequence, with the latter holding greater weight; therefore, Property/Efficacy Compatibility is the most commonly used method, while it also relies on the foundational medicinal properties of taste, property, and post-digestive taste. The Tibetan medical theoretical system is rooted in the doctrines of the “Five Sources” (འབྱུང་བ་ལྔ། Earth ས, Water ཆུ, Fire མེ, Wind རླུང་, Space ནམ་མཁའ) and the “Three Nyépa” (ཉེས་པ་གསུམ། rLung རླུང་, mKhris-pa མཁྲིས་པ, Bad-kan བད་ཀན). It posits that the “Five Sources” form the basis of all things, and medicines are also endowed by them. The combinations of Earth-Water, Fire-Earth, Water-Fire, Water-Wind, Fire-Wind, and Earth-Wind successively generate the “Six Tastes” (རོ་དྲུག Sweet མངར, Sour སྐྱུར, Salty ལན་ཚྭ, Bitter ཁ, Pungent ཚ, Astringent བསྐ) of medicines. The Six Tastes transform through digestion to form the “Three Post-Digestive Tastes” (ཞུ་རྗེས་གསུམ། Sweet མངར, Sour སྐྱུར, Bitter ཁ). From the Six Tastes and their Five Sources attributes are derived the “Eight Properties” (ནུས་པ་བརྒྱད) through which medicines act on the human body: Heavy (ལྕི), Unctuous (སྣུམ), Cool (བསིལ), Dull (རྟུལ), Light (ཡང་), Rough (རྩུབ), Hot (ཚ), and Sharp (རྣོ). Among these, Heavy and Unctuous properties treat rLung disorders; Cool and Dull properties treat mKhris-pa disorders; Light, Rough, Hot, and Sharp properties treat Bad-kan disorders. These Eight Properties are further concretely elaborated into 17 therapeutic effects, known as the “Seventeen Potencies” (ཡོན་ཏན་བཅུ་བདུན། Soft, Heavy, Warm, Unctuous, Stable, Cold, Dull, Cool, Soft, Fluid, Dry, Drying, Hot, Light, Sharp, Rough, Mobile), which precisely counteract the 20 Pathological Characteristics (མཚན་ཉིད་ཉི་ཤུ) arising from the imbalance of the Three Nyépa (Rough, Light, Cold, Hard, Subtle, Mobile; Unctuous, Sharp, Hot, Light, Malodorous, Purgative, Wet; Unctuous, Cool, Heavy, Dull, Soft, Slimy, Stable). Meanwhile, Property/Efficacy Compatibility is based on taste, property, and post-digestive taste, further focusing on the holistic efficacy and therapeutic direction of medicines. It involves combining medicines according to their overall efficacy and classification (e.g., medicines for rLung disorders), making it the most commonly used and representative method in Tibetan clinical practice (Gongbu, 1985). Additionally, Tibetan medicine places great emphasis on the “Inherent Efficacy” (ངོ་བོའི་ནུས་པ) of medicines, i.e., their eight essential efficacies derived from their unique odor, place of origin, color, and form (homologous form). These characteristics serve as the core theoretical basis guiding the entire process of Tibetan medicine, from origin identification, collection, and processing to the analysis of medicinal properties, toxicity, and the determination of dosage and compatibility. In terms of formula compatibility structure, specific medicines are organized according to their roles within the whole, forming a synergistic system with the Monarch (King) Drug as the main medicine, assisted by Minister, Queen, Prince, Envoy, General, Soldier, Horse, Shield, Spear, Artisan, and other auxiliary drugs. This structure is similar to the “Monarch, Minister, Assistant, Envoy” system of Traditional Chinese Medicine (Wen et al., 2019), together constituting a therapeutic whole that achieves synergistic effects and balances the Three Nyépa.

Analyzed according to the “Taste, Property, Post-Digestive Taste” medicinal property theory, the tastes of the ingredients in RYZBF belong to the Six Tastes, with the primary tastes dominated by Sweet taste (33%) and Astringent taste (32%) (Zhuoma Cuo et al., 2023). The Sweet taste is generated by the “Earth and Water” of the Five Sources, possessing properties such as Heavy, Stable, Soft, Unctuous, Fluid, Dull, Dry, Cool, Soft, enabling it to counteract the Light, Shaking, Rough characteristics of rLung disorders and the Malodorous, Sharp, Purgative-Wet, Hot, Light characteristics of mKhris-pa. The Astringent taste is generated by “Earth and Wind,” possessing Dull, Cool, Dry properties, enabling it to counteract the Sharp, Hot, Unctuous, Purgative-Wet characteristics of mKhris-pa disorders. After digestion, the post-digestive tastes are categorized into Warm, Cool, and Neutral. In this formula, medicines with a Cool post-digestive taste constitute 50%, directly targeting mKhris-pa heat syndrome. Medicines with a Warm post-digestive taste constitute 33%, used to balance the cold nature of rLung and prevent excessive heat-clearing from inducing rLung disorders.

Analyzed according to traditional Tibetan medicinal efficacy classification, this formula, in addition to including medicines such as *Margaritifera, L. brachystachya,* and *Glycyrrhiza uralensis* traditionally described as having mind-awakening, orifice-opening, and nerve-nourishing properties, also employs classical combination principles documented in Tibetan medical texts, including the “Three Fruits,” (འབྲས་བུ་གསུམ) “Six Excellent Medicines,” (བཟང་པོ་དྲུག) “Three Serous-Fluid-Drying Medicines,” (ཆུ་སེར་སྨན་གསུམ) “Three Diuretic Medicines,” (ཆུ་སྨན་རྣོན་པོ་གསུམ) and “Three Pungent Medicines” (ཚ་བ་གསུམ) (Tenzin Phuntsok, 2005). Among them, the “Three Fruits” (Terminalia chebula, Terminalia bellirica, Phyllanthus emblica) harmonize the Three Nyépa and provide nourishment. The “Six Excellent Medicines” (Tabasheer, *C. sativus, Syzygium aromaticum, M. fragrans, Amomum kravanh, Amomum tsao-ko*) sequentially address the functions of the lungs, liver, life channel, heart, kidneys, and spleen, representing a common compatibility for nourishing the five viscera and harmonizing the Three Nyépa. Multiple medicines, primarily the “Three Serous-Fluid-Drying Medicines” (Succinum, *Cassia obtusifolia, Abelmoschus moschatus*), have the effect of drying serous fluid. The “Three Diuretic Medicines” (*Eriocheir sinensis, Lygodium japonicum*) promote diuresis and tonify the kidneys. The “Three Pungent Medicines” (*Alpinia officinarum, P. longum,* etc.) have the efficacy of enhancing stomach fire and promoting medicine absorption. Simultaneously, medicines like *Margaritifera* and *L. brachystachya* possess homologous form efficacy due to their special structure and shape. This embodies a balanced philosophy of harmonizing medicinal properties and comprehensive treatment. The entire formula, through the combination of medicines with diverse efficacies, collectively achieves the overarching therapeutic goal of “harmonizing the Three Nyépa.”

**FIGURE 1:**
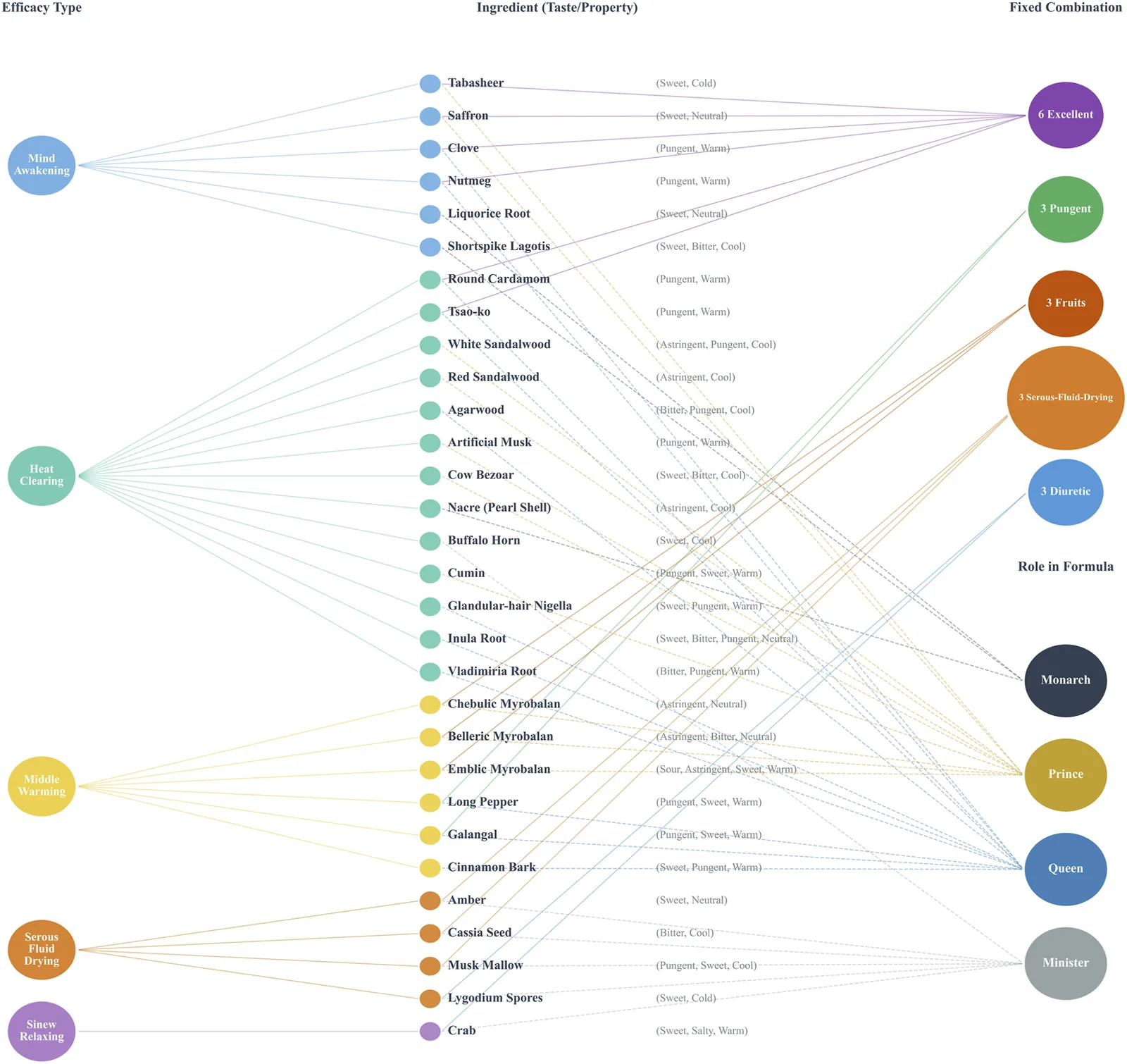
Compatibility structure and medicinal taste/property diagram of the Ruyi Zhenbao Formula. The diagram illustrates the hierarchical relationships among efficacy categories (left panel), individual ingredients with taste and property annotations (center), fixed classical combinations (upper right), and therapeutic roles—Monarch, Prince, Queen, and Minister—in the formula (lower right).

Analyzed according to compatibility structure based on Tibetan medicinal theory, RYZBF is composed of principal and auxiliary drugs. In this formula, Margaritifera (Concha Margaritifera), Lagotis brachystachya (Herba Lagotis Brachystachyae), and Glycyrrhiza uralensis (Radix Glycyrrhizae) are designated as Monarch Drugs within the Tibetan medicinal compatibility system, traditionally attributed with mind-awakening, orifice-opening, channel-dredging, and White Channel Disorder-treating properties. Eleven ingredients—*A. sinensis, C. cassia, M. fragrans, Amomum kravanh, Nigella glandulifera, S. aromaticum, Amomum tsao-ko, A. officinarum, P. longum* (part of the Three Pungent Medicines), *I. racemosa*, and *Vladimiria souliei*—are Queen Drugs. They primarily function as “kha-nus” (ཁ་གནོན། strengthening) medicines for rLung and rLung-mKhris-pa combined disorders, mainly treating rLung-mKhris-pa disorders, warming the stomach, tonifying the kidneys, and promoting medicine absorption. *Pterocarpus santalinus*, *Santalum album*, Tabasheer, *C. sativus, T. chebula, T. bellirica, Phyllanthus emblica, Cuminum cyminum,* Calculus Bovis, and *Moschus* are Prince Drugs, all clearing heat and detoxifying, and harmonizing the Three Nyépa. Succinum, *Cassia obtusifolia*, *A. moschatus* (the Three Serous-Fluid-Drying Medicines), *E. sinensis, L. japonicum* (the Three Diuretic Medicines), Cornu Bubali, and others are Minister Drugs, absorbing serous fluid and pus-blood. Combined, all medicines work together to achieve the effects of harmonizing the Three Nyépa, awakening the mind and opening the orifices, dredging the channel network, drying serous fluid, clearing chronic heat, and treating White Channel Disorder (characterized by heat, rLung disorder, serous fluid accumulation, and channel obstruction) (Zhuoma Cuo et al., 2023). In summary, the formula compatibility analysis discussed herein not only comprehensively elucidates the formulation principles of traditional Tibetan medical theory but also interprets, from a modern scientific perspective, the multi-component synergistic mechanisms underlying these traditional medicinal combinations. The medicinal properties and compatibility structure of the formula, as detailed in Table 1 and illustrated in Figure 1.

## 4 Pharmacological effects and underlying mechanisms

Modern research indicates that RYZBF contains various active metabolites, with flavonoids and their glycosides constituting the largest proportion (Chen et al., 2024; Xu et al., 2024). Pharmacokinetic studies have confirmed that multiple metabolites can be effectively absorbed and distributed to brain tissue (Hou et al., 2024a; Hou et al., 2024b; Hou et al., 2022), providing insights into potential mechanisms of action in central nervous system conditions. Pharmacological studies have preliminarily demonstrated that this formula possesses multiple effects including neuroprotection, anti-inflammation, analgesia, and microcirculation improvement, which align with the Tibetan medical principles of clearing heat, awakening the mind and opening the orifices, relaxing sinews and dredging collaterals, drying serous fluid, and treating White Channel Disorder. This multi-target modulatory action represents the modern scientific embodiment of the Tibetan medical concept of “holistic regulation.”

### 4.1 Chemical metabolites

Recent years have witnessed significant progress in phytochemical research on the Ruyi Zhenbao Formula, encompassing *in vitro* metabolite identification, analysis of blood-borne and brain-penetrating metabolites, pharmacokinetic profiling, and establishment of quality control methods. For *in vitro* metabolite identification, Chen et al. (2024). Employed UHPLC-Q Exactive Orbitrap HRMS to identify 126 metabolites from the Ruyi Zhenbao Pills, confirming the presence of multiple chemical classes including flavonoids, alkaloids, terpenoids, and organic acids. Utilizing a multi-dimensional “efficacy-target- metabolite-pharmacokinetics” association strategy, Xu et al. (2024) screened 40 candidate targets with strong intervention efficacy against neuropathic pain and identified 10 corresponding potential effector metabolites, suggesting that glycyrrhizic acid, gallic acid, and apigenin-7-O-glucuronide may serve as primary active substances. Regarding brain-penetrating metabolites, Hou et al. (2024b) discovered 13 metabolites capable of entering brain tissue, among which five—including daidzin, cholic acid, and piperine—were verified using reference standards, indicating that active metabolites from medicinal materials such as *Dalbergia odorifera, P. longum, G. uralensis,* and *C. cassia* can reach target organs to exert therapeutic effects. Pharmacokinetic studies demonstrated that agarotetrol, isoliquiritigenin, piperine, and ferulic acid can be absorbed and distributed to brain, heart, liver, and kidney tissues, with exposure levels positively correlated with dosage (Hou et al., 2024a; Hou et al., 2022). Furthermore, Han et al. (2017) and Xu and Xh (2020) established multi-index TLC identification and HPLC quantification methods, providing methodological foundations for quality control. Representative studies are summarized in Table 2.

Nevertheless, certain limitations persist in current research: most studies have focused on targeted tracking of known metabolites, lacking systematic non-targeted fingerprinting investigations; quantitative analyses have primarily addressed a limited number of marker compounds, failing to comprehensively reflect the overall chemical profile of the formula; and the pharmacodynamic relevance of brain-penetrating metabolites requires further validation. Variability in analytical methodologies across studies also limits comparability of results. In particular, the brain-penetrating metabolite studies were all conducted under normal physiological conditions, whereas in pathological states such as White Channel Disorder, blood-brain barrier (BBB) permeability is altered (e.g., increased due to inflammation or ischemia), which may affect the composition and concentration of metabolites entering brain tissue. Therefore, the current brain-penetrating profile may not fully reflect the clinical scenario, and future studies should validate brain-penetrating metabolites under disease-specific conditions (e.g., using MCAO or neuropathic pain models) to better inform pharmacodynamic mechanisms. These deficiencies represent critical areas requiring focused attention in future research (see Section 7.4 for detailed discussion).

### 4.2 Neuroprotective and repair effects

RYZBF has demonstrated multi-layered neuroprotective effects in preclinical studies. In a middle cerebral artery occlusion (MCAO) model induced by the suture method, studies established sham-operated, model (administered 0.5% sodium carboxymethyl cellulose solvent), and low-, medium-, and high-doseRYZBF groups (0.2, 0.4, 0.8 g/kg). Results after 14 days of consecutive intragastric administration post-surgery showed that, compared with the model group, medium and high doses (0.4, 0.8 g/kg) of RYZBF significantly improved neurological behavioral scores (*P <* 0.05) and significantly increased the number of neuronal nuclei antigen (NeuN)-positive neurons and the density of von Willebrand factor (vWF)-positive microvessels in the ischemic hemisphere (*P <* 0.05). Mechanistic studies indicated this effect was associated with the upregulation of brain-derived neurotrophic factor (BDNF), nerve growth factor (NGF), and vascular endothelial growth factor (VEGF) expression (*P <* 0.05) (Wang et al., 2016). While this study provides robust *in vivo* evidence, the absence of positive controls limits direct comparison with standard therapies. Concurrently, the formula significantly reduced brain edema (P < 0.05), ameliorated Nissl body damage, and regulated the protein expression of NF-κB p65, Bax, and Bcl-2 (*P <* 0.05) (Liu et al., 2015a). Another MCAO study further confirmed that RYZBF could upregulate the anti-apoptotic protein Bcl-2 in the cerebral cortex (*P <* 0.01), downregulate the pro-apoptotic proteins Bax, Cyt-C, and cleaved caspase-3 (*P <* 0.01), and simultaneously reduce the cerebral infarction rate (*P <* 0.01) (Luo et al., 2023). Though Luo’s combined use of RYZBF with Baimai Ointment makes it difficult to isolate the formula’s individual contribution. Furthermore, research on neurovascular unit repair demonstrated that RYZBF significantly promoted the expression of angiogenesis- and neuroregeneration-related factors such as CD34, VEGF, and GAP-43 in the ischemic area of a rat model of cerebral ischemia-reperfusion (*P <* 0.05) (Li et al., 2018).

The protective effects of RYZBF have been further validated in other models. At the cellular level, a glutamate-induced PC12 cell injury model was established, including normal control, model, and different concentration RYZBF treatment groups. MTT assay detection revealed that, compared with the model group, the RYZBF treatment groups showed significantly increased cell viability (*P <* 0.05), significantly reduced lactate dehydrogenase leakage rate (*P <* 0.05), and significantly enhanced superoxide dismutase activity (*P <* 0.05) (Yan and Wang, 2016). In a zebrafish central nerve injury model, RYZBF not only showed high protection rates against central nerve and axonal injury (both *P <* 0.05) but also significantly promoted peripheral motor nerve regeneration (93%, *P <* 0.001) and remyelination (41%, *P <* 0.001), demonstrating potent nerve repair-promoting capability (Zhu et al., 2017). Regarding cognitive function improvement, a study using a vascular dementia rat model indicated that RYZBF significantly improved learning and memory abilities (*P <* 0.01) and increased the levels of various monoamine neurotransmitters in the brain (*P <* 0.05) (Chen et al., 2016). Collectively, these studies provide multi-level evidence for neuroprotection, but the lack of chemical characterization of preparations across most studies limits reproducibility and translational value.

### 4.3 Anti-inflammatory and immunomodulatory effects

RYZBF exerts its anti-inflammatory effects through the regulation of complex inflammatory factor networks. In a papain-induced rat osteoarthritis (OA) model, RYZBF (0.45, 0.9 g/kg) alleviated joint swelling and pain. Its mechanism is associated with promoting the synthesis of type II collagen (COL II), inhibiting the upregulation of osteopontin (OPN), and modulating the balance of inflammation-related biomarkers such as MMP1, TNF-α, COX2, IL-1β, and iNOS in joints or serum (Li et al., 2023). Providing a valuable example of metabolite-activity relationship research. However, the absence of full fingerprint analysis and exclusive use of male rats limit the comprehensiveness of these findings. In a lipopolysaccharide-induced ATDC5 cell inflammation model, qRT-PCR results showed that RYZBF significantly downregulated the mRNA expression of pro-inflammatory factors such as TNF-α, iNOS, COX-2, and IL-6 (*P <* 0.05) (Zhou et al., 2024). In a post-stroke central pain model, immunofluorescence analysis showed that RYZBF significantly inhibited CXCL16 expression (*P <* 0.05) and the overactivation of microglia (Huang et al., 2024). Regarding the regulation of inflammatory factor balance, enzyme-linked immunosorbent assay results indicated that RYZBF effectively modulated the pro-inflammatory/anti-inflammatory cytokine balance. Compared with the model group, RYZBF significantly reduced the expression of the pro-inflammatory factors IL-1α and CCL5 (*P <* 0.05) while elevating the levels of the anti-inflammatory factors IL-4 and G-CSF (*P <* 0.05) (Jia et al., 2022).

In terms of immune cell function regulation, a hemorrhagic stroke model study revealed that RYZBF improved neurological injury (*P <* 0.05) and central pain symptoms (*P <* 0.05), and reduced the number of CX3CR1-Ly6C + labeled classical monocytes (*P <* 0.01) (Wang et al., 2024). In neurogenic inflammation regulation research, immunohistochemistry results showed that RYZBF significantly decreased NGF and TRPV1 protein expression (*P <* 0.05) (Li et al., 2020). Multi-model validation studies further support its anti-inflammatory efficacy. A zebrafish inflammation model showed that RYZBF inhibited neutrophil aggregation in a dose-dependent manner (*P <* 0.05) (Du et al., 2017). Classical inflammation experiments confirmed its significant inhibitory effects on xylene-induced mouse ear swelling and egg white-induced rat paw edema (*P <* 0.01) (Song et al., 2018). In a chronic constriction injury model of the sciatic nerve, the formula reduced serum IL-10 and TNF-α content (*P <* 0.05) (Huang et al., 2019). These studies collectively indicate that RYZBF modulates inflammation through multiple pathways, though the predominance of single-pathway investigations and lack of standardized preparations warrant more systematic approaches in future research.

The “drying serous fluid” efficacy in Tibetan medicine is not a separate pharmacological mechanism but a traditional description of the clinical consequences of the anti-inflammatory and anti-exudative actions detailed above. RYZBF reduces joint swelling, neutrophil aggregation, and pro-inflammatory cytokines (TNF-α, IL-1β, COX-2), all of which resolve what Tibetan medicine terms “serous fluid” (inflammatory exudate). Ingredients primarily associated with this effect, such as Succinum, Cassia obtusifolia, Abelmoschus moschatus, among others, likely act through these pathways. While direct evidence on fluid transporter regulation (e.g., aquaporins) or lymphatic drainage is lacking, we hypothesize these mechanisms may be involved and warrant future investigation.

### 4.4 Multi-mechanism analgesic effects

RYZBF has shown analgesic effects in preclinical models through multiple pain signaling pathways. In an osteoarthritis pain model, research results showed that RYZBF significantly inhibited static and dynamic mechanical allodynia (*P <* 0.05), decreased miniature excitatory postsynaptic current (mEPSC) frequency (*P <* 0.05), and inhibited the phosphorylation of the GluN1 subunit of NMDA receptors in the spinal dorsal horn (*P <* 0.05) (Chen et al., 2022), revealing its analgesic mechanism at the molecular level. In migraine model studies, real-time quantitative polymerase chain reaction analysis showed that RYZBF significantly downregulated the expression of multiple pain-related genes (*P <* 0.05) (Zhou et al., 2020); electroencephalogram studies indicated the formula clearly inhibited pathological EEG changes (*P <* 0.05), showing particular sensitivity in modulating theta band waves (Luo et al., 2018a), suggesting the drug alleviates migraine attacks by regulating the PKA-CREB signaling pathway, mediating changes in PACAP and PAC1 content. In central pain model studies, behavioral test results showed that RYZBF significantly improved pain-related behaviors (*P <* 0.01) (Jia et al., 2022). A zebrafish pain model study confirmed the formula possesses both peripheral and central analgesic effects (*P <* 0.05) (Du et al., 2017). In neuropathic pain research, hot plate test latency detection showed that RYZBF significantly prolonged latency (*P <* 0.05) (Huang et al., 2019); mechanistic studies further confirmed that the drug can dose-dependently alleviate mechanical allodynia and cold/heat stimulus-induced pain in spinal nerve ligation (SNL) mice by inhibiting the CXCL10-CXCR3 signaling pathway and the expression of its downstream molecules in the spinal dorsal horn, with transcriptomics and molecular docking suggesting its active ingredients have strong affinity for the target protein CXCL10 (Yang et al., 2024). An innovative approach that now requires experimental validation of the predicted interactions. Furthermore, studies found the drug could inhibit neurogenic inflammatory pain hypersensitivity by reducing the upregulating effect of NGF and TrkA on TRPV1 (*P <* 0.05) (Li et al., 2020). In an acetic acid-induced writhing test, the formula significantly inhibited writhing responses in mice (*P <* 0.01) (Song et al., 2018). These multi-pathway mechanisms together constitute its complex analgesic network. These findings reveal a complex analgesic network, though future studies should integrate multi-omics approaches and functional validation to fully elucidate synergistic mechanisms.

### 4.5 Blood circulation improvement and antioxidant effects

RYZBF has significant effects on the circulatory system and antioxidative stress. Hemorheology tests showed the formula improved multiple hemorheological parameters (*P <* 0.05), with a particularly notable effect on reducing hematocrit values, indicating that its function of activating blood and resolving stasis is closely related to altering hemorheological status (Luo et al., 2018b). Additionally, in a rat MCAO model, the formula significantly increased serum superoxide dismutase (SOD) activity, reduced malondialdehyde (MDA) content, and decreased lactate dehydrogenase (LDH) activity (*P <* 0.05), confirming its ability to alleviate cellular oxidative damage (Xie et al., 2014). In studies on cellular oxidative damage protection mechanisms, MTT assay detection further showed that RYZBF significantly increased cell viability, reduced nitric oxide content, and enhanced SOD activity (all *P <* 0.05), clarifying its antioxidant and cytoprotective effects at the *in vitro* level (Yan and Wang, 2016).

Other research indicates the formula exhibits synergistic therapeutic effects in combination therapy. The combined use of Ershiwuwei Shanhu Pill, RYZBF, and Ershiwei Chenxiang Pill exerted protective effects on ischemic brain tissue by reducing the infarction area (*P <* 0.05), alleviating brain edema (*P <* 0.05), and inhibiting oxidative stress reactions, among other pathways (Xie et al., 2014). The mechanisms by which Tibetan “Zhenbao” category compound formulas improve central nervous system diseases primarily involve reducing inflammatory responses, decreasing oxidative stress damage, and inhibiting neuronal apoptosis, among other aspects (Luo et al., 2022). While these studies provide preliminary evidence, the indirect nature of most assessments and the inability to isolate RYZBF’s individual contribution in combination therapies highlight the need for more direct investigations of vascular function and formula-specific effects. The core mechanisms and their details are presented in Table 3.

**TABLE 3:** Core pharmacological action mechanisms and experimental evidence for RYZBF.

Action category	Core mechanism/target	Experimental model	Key results (vs. model group)	References
Neuroprotection and repair	Promotes neurovascular repair: upregulates BDNF, NGF, VEGF	Rat cerebral ischemia/reperfusion model	Neurological function score ↑; Number of NeuN + neurons and vWF + vessels in ischemic hemisphere ↑; BDNF, NGF, VEGF levels in brain tissue ↑ (all *P <* 0.05)	Wang et al. (2016)
	Anti-apoptosis: regulates Bcl-2/Bax/Caspase-3 pathway	Rat MCAO model (suture method)	Cerebral infarction rate ↓, neurological score ↑; Bax, Cyt-C, cleaved Caspase-3 ↓, Bcl-2 ↑ (all *P <* 0.01)	Liu et al., (2015a) ; Luo et al. (2023)
	Promotes vascular and neural regeneration: upregulates CD34, VEGF, GAP-43	Rat cerebral ischemia/reperfusion model	Positive expression of CD34, VEGF, GAP-43 in ischemic hemisphere on postoperative day 7 ↑ (*P <* 0.05)	Li et al. (2018)
	Promotes axon and myelin regeneration	Mycophenolate mofetil-induced zebrafish nerve injury model	Central nerve injury protection rate 50%, axonal injury protection rate 48% (*P <* 0.001, *P <* 0.05); Peripheral nerve regeneration rate 93%, myelin regeneration rate 41% (both *P <* 0.001)	Zhu et al. (2017)
	Improves cognitive function: regulates brain monoamine neurotransmitters	Vascular dementia rat model (bilateral common carotid artery ligation)	Correct choices in Y-maze ↑; Dopamine, norepinephrine, 5-hydroxytryptamine in brain ↑ (*P <* 0.05)	Chen et al. (2016)
	Cytoprotection: Reduces excitotoxicity, increases antioxidant enzyme activity	Glutamate-induced PC12 cell injury model	Cell viability ↑; Lactate dehydrogenase leakage rate and nitric oxide ↓; Superoxide dismutase activity ↑ (all *P <* 0.05)	Yan and Wang (2016)
Anti-inflammatory and immunomodulation	Modulates joint inflammatory network and cartilage metabolism: Inhibits TNF-α, COX2, IL-1β, iNOS, MMP1; promotes COL II synthesis, inhibits OPN upregulation	Papain-induced rat knee osteoarthritis (OA) model	Joint swelling and pain significantly alleviated (*P <* 0.05); Levels of pro-inflammatory factors and degradative enzymes in serum and joint tissues ↓, cartilage matrix COL II expression ↑	Li et al. (2023)
	Inhibits classical inflammatory pathways: downregulates TNF-α, iNOS, COX-2, IL-6 mRNA	LPS-induced ATDC5 chondrocyte inflammation model	TNF-α, iNOS, COX-2, IL-6 mRNA expression ↓ (*P <* 0.05)	Zhou et al. (2024)
	Modulates neuroimmunity: inhibits microglial activation and CXCL16 expression	Mouse central post-stroke pain (CPSP) model	Spinal inflammation ↓, pain behavior improved; CXCL16 expression in high-dose group ↓ (*P <* 0.05)	Huang et al. (2024)
	Balances inflammatory network: modulates pro-inflammatory (IL-1α, CCL5)/anti-inflammatory (IL-4, G-CSF) factors	Mouse central pain post-thalamic hemorrhage model	IL-1α, CCL5 ↓; IL-4, G-CSF, Ang-2 ↑ (*P <* 0.05 or *P <* 0.01)	Jia et al. (2022)
Multi-mechanism synergistic analgesia	Inhibits spinal CXCL10-CXCR3 signaling axis: downregulates CXCL10, CXCR3 and downstream molecules	Mouse spinal nerve ligation (SNL) neuropathic pain model	Dose-dependently alleviates mechanical allodynia, cold and heat stimulus-induced pain (*P <* 0.05); CXCL10, CXCR3 protein expression in spinal dorsal horn significantly ↓; Intrathecal rmCXCL10-induced hyperalgesia reversed	Yang et al. (2024)
	Inhibits central sensitization: reduces spinal dorsal horn neuron excitability (p-NMDAR ↓, mEPSCs frequency ↓)	Mouse osteoarthritis (DMM) pain model	Tactile allodynia ↓; mEPSCs frequency ↓, pS897-GluN1 phosphorylation in spinal dorsal horn ↓ (*P <* 0.05)	Chen et al. (2022)
	Modulates pain-related signaling pathways: mediates PKA-CREB pathway, affecting PACAP/PAC1	Nitroglycerin-induced rat migraine model	Serum PACAP, PAC1 protein and midbrain PACAP, AC, CREB, PKA mRNA ↓ (*P <* 0.05); EEG improvement	Zhou et al. (2020)
	Inhibits peripheral sensitization (neurogenic inflammation): downregulates NGF, TrkA, TRPV1	Nitroglycerin-induced rat migraine model	Serum and midbrain NGF, TrkA, TRPV1 protein and gene expression ↓ (*P <* 0.05 or *P <* 0.01)	Li et al. (2020)
Antioxidant and circulation improvement	Reduces oxidative stress: increases SOD activity, decreases MDA content and LDH activity	Rat MCAO model (Combination: Ershiwuwei Shanhu Pill + RYZBF + Ershiwei Chenxiang Pill)	Serum SOD ↑, MDA ↓, LDH ↓ (*P <* 0.05)	Xie et al. (2014)
	Improves hemorheology	Acute blood stasis rat model (adrenaline + ice-water swimming)	Whole blood viscosity, plasma viscosity, hematocrit, fibrinogen ↓ (*P <* 0.05)	Luo et al. (2018b)

## 5 Clinical application research

The efficacy of RYZBF has been evaluated in high-quality clinical studies, including large-scale, multicenter randomized controlled trials (RCTs) and systematic reviews with meta-analyses. Multiple studies confirm that this medication has significant therapeutic effects in several clinical fields, such as osteoarthritis, cerebrovascular diseases, and neurological disorders, effectively improving patients’ clinical symptoms, signs, and quality of life. Safety evaluations indicate a low incidence of adverse reactions and good clinical application safety. These studies not only validate the clinical value of the drug from a modern medical perspective, but its multi-system, multi-target therapeutic characteristics also fully align with the core concepts of holistic regulation of the Three Nyépa and syndrome differentiation in the Tibetan medical theory of “White Channel Disorder.”

### 5.1 High-level evidence for musculoskeletal system diseases

The following multicenter randomized controlled trials (RCTs) and meta-analyses provide the highest level of evidence. In the treatment of osteoarthritis (OA), a multicenter, randomized, double-blind, placebo-controlled clinical trial involving 240 patients showed that RYZBF was significantly superior to the control group in improving total WOMAC score and pain score for patients with initial and early-stage knee OA. Marginal model analysis results indicated that the observation group was better than the control group in improving total WOMAC and pain scores, with statistically significant differences (*P <* 0.05) (Sun et al., 2024). Notably, subgroup analysis suggested enhanced efficacy in patients aged 56–65 and those with baseline VAS scores of 4–5, indicating the need for further stratified studies. Subgroup analysis found that the efficacy was particularly significant for patients with baseline VAS scores of (4, 5) and those aged 56–65. Its mechanism of action is closely related to regulating joint inflammatory response and inhibiting chondrocyte apoptosis (Zhou et al., 2024). In rheumatoid arthritis treatment, the total effective rate of RYZBF monotherapy was significantly better than that of Total Glucosides of Peony Pill (*P <* 0.05) (Mimaciren, 2017), and its combination with Total Glucosides of Peony Capsules also showed better clinical efficacy (*P <* 0.05) (Zhai, 2015). Though the lack of blinding in these studies limits evidence strength. Clinical comprehensive evaluation indicates that RYZBF has good safety, reasonable economic efficiency, and good clinical application value for treating OA (Zhang et al., 2023).

In the field of gouty arthritis treatment, studies show that the 3-day effective rate of RYZBF monotherapy for acute gouty arthritis reached 96.7%, with joint swelling-pain index reduced by 94.8% and serum uric acid levels reduced by 91.2%; the 7-day effective rate was 100%, with no observed liver or kidney function damage (Cai, 2015). A network meta-analysis showed that among various Chinese patent medicine regimens for treating gouty arthritis, the combination of RYZBF with chemical drugs had the lowest incidence of adverse reactions (Li et al., 2021). A clinical retrospective analysis indicated that the combination of RYZBF with Chinese herbal medicine for acute gouty arthritis achieved an effective rate as high as 91.7%, significantly higher than the 75% in the NSAID control group (*P <* 0.05). This treatment regimen effectively reduced serum uric acid and C-reactive protein levels with a low incidence of adverse reactions (Song J. H. et al., 2022).

In the treatment of other musculoskeletal diseases, patients with lumbodorsal myofasciitis treated with RYZBF combined with fire needle therapy achieved a total effective rate of 98.67%, significantly better than the 86.67% in the fire needle monotherapy group (*P* = 0.005). The combination therapy significantly improved VAS scores, Japanese Orthopaedic Association (JOA) assessment scores, and Barthel Index, enhancing patients’ quality of life (Ye and Lin, 2023). In osteoporosis treatment, the combination of zoledronic acid, RYZBF, and alfacalcidol tablets achieved an effective rate of 91.94%, significantly better than monotherapy (*P <* 0.05), and significantly increased bone mineral density (Xu and Cheng, 2016). For chronic musculoskeletal pain related to White Channel Disorder, RYZBF treatment achieved a total effective rate of 92.39%, significantly reducing VAS scores and improving various limb function indices (Song M. et al., 2022). While high-quality RCT evidence supports RYZBF’s efficacy in OA, studies in other musculoskeletal conditions are predominantly small-scale or observational, warranting cautious interpretation.

### 5.2 Mixed-level evidence for cerebrovascular diseases (RCTs and observational studies)

This section includes both high-quality RCTs and observational studies; the level of evidence varies, and the highest available level is indicated for each study. For acute-phase cerebral infarction treatment, a multicenter, multi-arm, randomized, double-blind trial targeting acute ischemic stroke (within 14 days of onset) further confirmed that RYZBF, used alone or combined with Baimai Ointment, significantly promoted long-term functional recovery in patients. At 90 days post-treatment, the improvement in Fugl-Meyer assessment scores in the combination group, RYZBF monotherapy group, and Baimai Ointment monotherapy group was significantly better than in the placebo group (mean differences: 12.02, 10.68, 10.05 points, all *P <* 0.001); the proportions of patients achieving functional independence (modified Rankin Scale score ≤2) and activities of daily living self-sufficiency (Barthel Index ≥95) were also significantly higher in the treatment groups (all *P <* 0.01), without increasing the risk of adverse events (Jiang et al., 2025; Qin et al., 2024). The combination therapy showed the greatest benefit, warranting further investigation into synergistic mechanisms. Combined sequential treatment of acute cerebral infarction with RYZBF and butylphthalide showed a total effective rate (85.0%) with no statistically significant difference from the control group (80.0%), but demonstrated advantages in regulating CRP expression and improving neurological function, limb function, and living ability (Li, 2025). For cerebrovascular vasospasm after subarachnoid hemorrhage, RYZBF combined with edaravone significantly improved GCS scores, Barthel Index, and NIHSS scores (*P <* 0.05) (Liu X. B. et al., 2015). Tibetan classical formula combination therapy at different time periods shows unique advantages in anti-cerebral ischemia, reducing oxidative stress damage and infarct volume (Liu et al., 2015b).

In the treatment of post-stroke sequelae, a double-blind, placebo-controlled trial involving patients in the stroke recovery period (15 days to 6 months post-onset) showed that adding RYZBF (2 g/dose, twice daily, for 4 weeks) to conventional rehabilitation significantly improved neurological function. After 4 weeks of treatment, the Fugl-Meyer Motor Function Score, Berg Balance Scale score, and Fugl-Meyer Sensory Assessment score in the RYZBF group were significantly better than those in the placebo group (all *P <* 0.05); at the 8-week follow-up, the advantages in balance function and activities of daily living (modified Barthel Index) were maintained (*P <* 0.05), with good safety (Ling et al., 2022). Research based on Hospital Information System (HIS) clinical case records found that RYZBF is one of the core formulas for treating Sa Zi Bu Disorder (post-stroke sequelae), with an application frequency of 322 times among 479 patients, often combined with Qishiwei Zhenzhu Pill and Ershiwei Chenxiang Pill, forming time-sequenced medication regimens (Gong et al., 2019). According to the Three Nyépa theory, Tibetan medicine employs different time-period combination regimens to treat Lung Zi Bu Disorder (ischemic stroke), with RYZBF typically scheduled for morning administration (Guo et al., 2016).

In the treatment of other cerebrovascular diseases, for vascular dementia, one study showed that RYZBF combined with oxiracetam significantly improved patients’ cognitive function and living ability. The total effective rate in the treatment group was 95.16%, significantly higher than the 83.87% in the control group (*P <* 0.05). After treatment, the MMSE and ADL scores in the observation group were significantly higher than in the control group, and the adverse reaction incidence (6.45%) was significantly lower than in the control group (19.35%) (*P <* 0.05) (Zhang and Li, 2021). Another study also confirmed that the total effective rate of this combination regimen (87.39%) was significantly higher than that of oxiracetam monotherapy (68.18%, *P <* 0.05), better improving MMSE and ADL scores while reducing serum CRP and NSE levels (Zhang et al., 2019). Though small sample sizes and lack of blinding limit evidence strength. Furthermore, studies indicate that Tibetan classical formulas for “Serous Fluid Disorder” can improve cognitive impairment in Alzheimer’s disease through the “metabolism-immune-central nervous system” network (He et al., 2024). Meanwhile, a review analysis focusing on the theory of “White Channel Disorder” further points out that RYZBF and other Tibetan “Zhenbao” category compound formulas have clear therapeutic advantages in various central nervous system diseases such as stroke, Alzheimer’s disease, epilepsy, Parkinson’s disease, vascular neurogenic headache, and vascular dementia (Huang and Xu, 2024, Luo et al., 2022). The high-quality RCTs in acute stroke and recovery provide robust evidence, but studies in other cerebrovascular conditions require larger, well-controlled trials for validation.

### 5.3 Observational and preliminary evidence for neurological disorders

The studies in this section are predominantly small-sample, non-blinded, or observational; their findings should be interpreted cautiously. In the treatment of neuropathic pain, a meta-analysis incorporating 8 clinical trials with a total of 1,327 patients showed that the effective rate of RYZBF for neuropathic pain was significantly better than that of the control group (OR = 5.07, 95% CI 3.56–7.23, *P <* 0.00001) (Xu et al., 2018). While this provides strong evidence, heterogeneity in the quality of included studies warrants cautious interpretation. For specific types of neuralgia, the total effective rate of RYZBF for trigeminal neuralgia was 78.16%, better than the 55.17% in the Western medicine control group (Zeng, 2019); its total effective rate for postherpetic neuralgia reached 95.35%, significantly better than the 77.5% in the mecobalamin control group (*P <* 0.05) (Zhao, 2016). Though these small, non-blinded trials require confirmation. Mechanistic studies indicate that the drug can alleviate neuropathic pain by regulating the expression of factors such as IL-1α, CCL5, IL-4, G-CSF, and Ang-2 (Jia et al., 2022).

For migraine treatment, clinical observation found that RYZBF significantly reduced the frequency, duration, and severity of migraine attacks, with efficacy superior to ergotamine caffeine and higher safety (Zhang, 2017). Electroencephalogram (EEG) studies showed that the drug could inhibit pathological EEG changes induced by nitroglycerin in a rat migraine model, such as decreased delta band percentage and increased alpha1, alpha2, theta, and beta band percentages, exhibiting good analgesic effects during migraine attacks (Luo et al., 2018a).

In the treatment of peripheral neuropathy, the combination of RYZBF and acyclovir for peripheral facial paralysis achieved a total effective rate (97.56%) significantly higher than that of acyclovir monotherapy (82.93%) (*P <* 0.05), significantly improving facial nerve conduction function and shortening the latency of BRR1 and R2 waves (Jia et al., 2022; R, 2023). A randomized controlled trial showed that adding RYZBF to conventional treatment more effectively improved facial nerve function and clinical symptoms in patients with peripheral facial paralysis (*P <* 0.05), promoting overall recovery (Zhoujie and Ga, 2022). In treating vincristine-induced peripheral neurotoxicity, the combination of RYZBF and mecobalamin achieved a total effective rate of 95%, significantly better than the 80% in the mecobalamin monotherapy group (*P <* 0.05) (Zhou, 2016).

In the treatment of other neurological disorders, female patients with pudendal neuralgia treated with RYZBF combined with warm needle acupuncture at the BaLiao points showed better efficacy than warm needle acupuncture alone, effectively alleviating pain, swelling, and other symptoms and improving lumbosacral vertebral function (Xiao and Tu, 2025). For hemiplegic shoulder pain, RYZBF combined with rehabilitation training significantly improved shoulder pain and joint range of motion (*P <* 0.05) and reduced recurrence rates (Yang, 2023; Niang, 2017). Patients with sciatica treated with oral Tibetan medicine combined with salt-heated compress therapy effectively relieved symptoms (Lalang Duojie, 2016), and postpartum low back pain patients treated with Tibetan medicine also achieved good results (Cuojimazhuaxi, 2014). In the treatment of diseases related to White Channel Disorder, Tibetan medical theory holds that White Channel Disorder encompasses various neurological diseases in modern medicine. As the preferred medication for White Channel Disorder, RYZBF is widely used clinically for various related diseases (Luo et al., 2017; Xi et al., 2017). The meta-analysis provides robust evidence for neuropathic pain, but studies on other neurological conditions need more rigorous designs to establish efficacy.

### 5.4 Exploratory evidence for other applications

This section summarizes exploratory, uncontrolled, or time-sequenced regimen studies; no high-level RCTs are currently available. Regarding combination treatment regimens, Tibetan medicine often employs different time-period combination regimens based on the Three Nyépa theory to treat Lung Zi Bu Disorder (ischemic stroke). Research found that combining Qishiwei Zhenzhu Pill (morning), Ruyi Zhenbao Pill (late morning), Sanshiqiwei Banmao Pill (noon), and Ershiwei Chenxiang Pill (evening) significantly improved neurological function and quality of life (Guo et al., 2016). This time-sequenced medication regimen embodies the wisdom of Tibetan temporal medicine, with an observed effective rate of 97.5% (Deng et al., 2022). Different time-period combinations of Tibetan classical formulas show synergistic effects in anti-cerebral ischemia, reducing infarct volume and inhibiting cell apoptosis (Liu et al., 2015b). These observational studies provide valuable insights into clinical practice patterns, though the lack of control groups limits causal inference.

**FIGURE 2:**
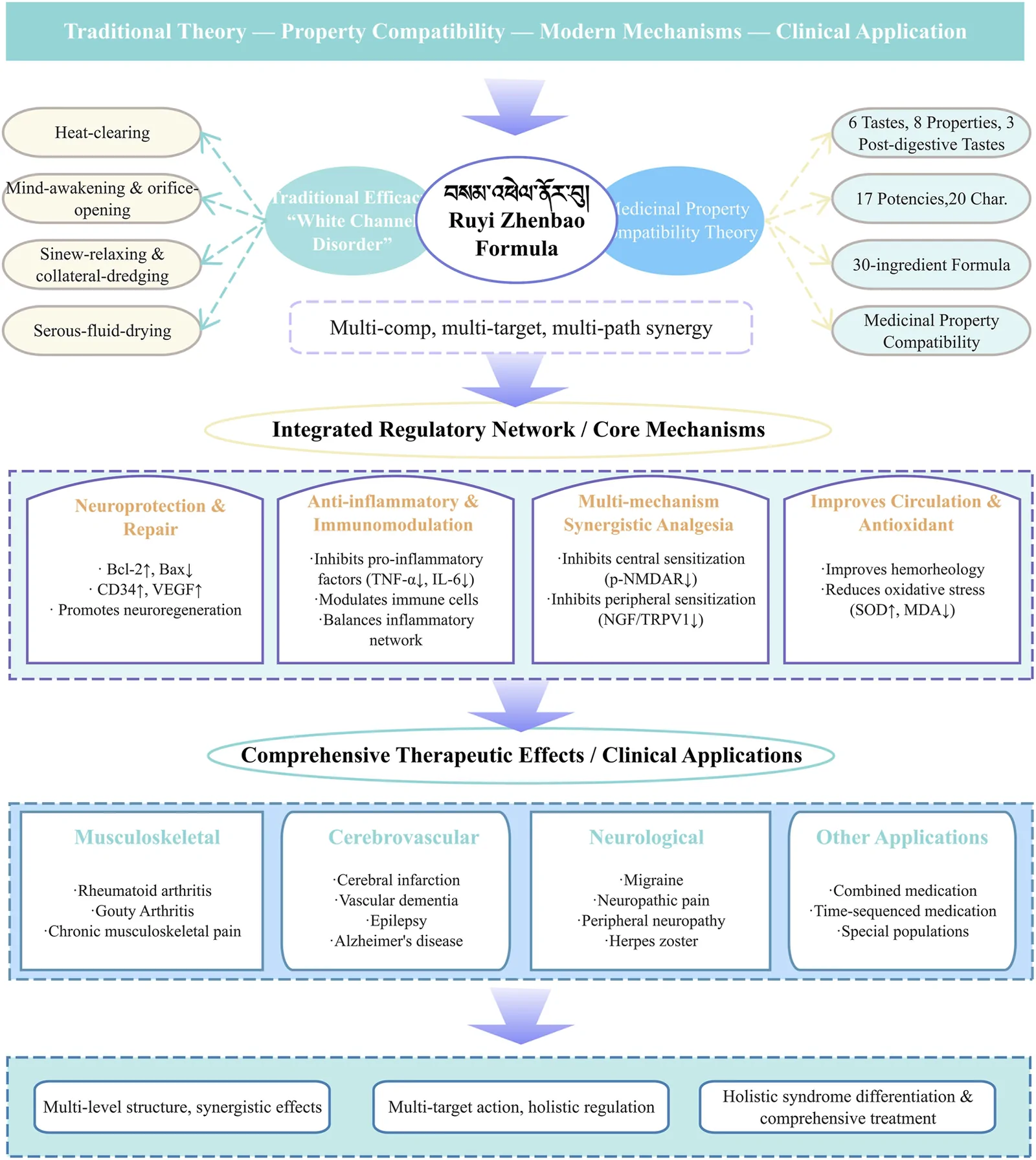
Integrative “Theory–Compatibility–Mechanisms–Application” Framework of RYZBF. Arranged from top to bottom, the schematic links the traditional Tibetan medical theory and medicinal-property compatibility (top) with the core pharmacological mechanisms (middle) and the clinical applications (bottom), illustrating the multi-target, holistic regulation of the formula.

Regarding application in special populations, the safety of RYZBF in elderly patients is good. Subgroup analysis showed that knee OA patients aged 56–65 treated with RYZBF had a significantly greater reduction in total TCM syndrome score than the control group (*P <* 0.05) (Sun et al., 2024). Analysis of the clinical application of ethnic medicines in medical institutions such as the Third Affiliated Hospital of Beijing University of Chinese Medicine shows that RYZBF is widely used in orthopedics-traumatology and encephalopathy departments with good rational use of medicine (Lu et al., 2017). Prospective trials are needed to validate the efficacy of time-sequenced regimens and establish evidence-based guidelines. The main clinical studies and outcomes are summarized in Table 4 The integrative schematic diagram of the theory, mechanisms, and application of RYZBF is shown in Figure 2.

**TABLE 4:** High-quality evidence-based medicine evidence for the clinical application of RYZBF.

Disease category	Specific indication	Study design	Sample size (T/C)	Intervention (treatment vs. control)	Primary efficacy outcome	References
Musculoskeletal	Knee OA (initial, early stage)	Multicenter, Randomized, Double-blind, Placebo-controlled Trial	120/120	RYZBF + Health education vs. placebo + health education	Improvement in total WOMAC score and pain score superior to control (*P <* 0.05)	Sun et al. (2024)
	Osteoarthritis	Clinical Comprehensive Evaluation	N/A	Comprehensive evaluation (“6 + 1” dimensions)	Safety Grade A, efficacy Grade B, comprehensive value category B	Zhang et al. (2023)
	Rheumatoid arthritis	RCT	240/240	RYZBF vs. total glucosides of peony pill	Total effective rate significantly superior in treatment group (*P <* 0.05)	Mimaciren (2017)
	Acute gouty arthritis	RCT	60/60	RYZBF + CHM vs. NSAIDs	Higher total effective rate (91.7% vs. 75.0%, *P <* 0.05); Superior reduction in serum UA and CRP (*P <* 0.05)	Song J. H. et al. (2022)
Cerebrovascular	Post-stroke motor/sensory dysfunction (recovery)	Randomized, double-blind, placebo-controlled trial	60/60	RYZBF + conventional Tx vs. placebo + conventional Tx	After 4 weeks, improvements in FMA-M, BBS, FMA-S scores superior to control (*P <* 0.05); Good safety.	Ling et al. (2022)
	Acute ischemic stroke	Multicenter, randomized, double-blind, placebo-controlled trial (4-arm)	RYZBF + BMO: 108/RYZBF: 108/BMO: 99/Placebo: 108	Oral RYZBF + Topical BMO Oral RYZBF only Topical BMO only vs. dual placebo	At 90 days, ΔFugl-Meyer score in all Tx groups > placebo (all *P <* 0.001). RYZBF + BMO showed greatest improvement.	Jiang et al. (2025), Qin et al. (2024)
	Acute cerebral infarction	RCT	40/40	RYZBF + butylphthalide sequential Tx vs. butylphthalide sequential Tx	Improvement in CRP, NIHSS, FMA, and ADL scores > control (*P <* 0.05)	Li (2025)
	Vascular dementia	RCT	111/110	RYZBF + oxiracetam vs. oxiracetam	Higher total effective rate (87.39% vs. 68.18%, *P <* 0.05); Superior improvement in MMSE and ADL (*P <* 0.05)	Zhang et al. (2019)
	Cerebral vasospasm after SAH	RCT	69/69	RYZBF + edaravone vs. edaravone	Higher clinical total effective rate (*P <* 0.05); superior improvement in neurofunction and ADL (*P <* 0.05)	Liu X. B. et al. (2015)
Neurological	Various neuropathic pain	Meta-analysis (8 RCTs)	Total 1,327 cases	RYZBF vs. control drugs	Efficacy superior to control [OR = 5.07, 95% CI (3.56, 7.23), *P <* 0.00001]	Xu et al. (2018)
	Trigeminal neuralgia	RCT	87/87	RYZBF vs. WM (Vit B12 + phenytoin Na, etc.)	Higher total effective rate (78.16% vs. 55.17%, *P <* 0.05)	Zeng (2019)
	Postherpetic neuralgia	RCT	43/40	RYZBF vs. mecobalamin	Significantly higher total effective rate (95.35% vs. 77.5%, *P <* 0.05)	Zhao (2016)
	Migraine	RCT	30/32	RYZBF vs. ergotamine and caffeine tablets	Superior in reducing attack frequency, duration, severity (*P <* 0.05); Higher safety	Zhang (2017)
​	Peripheral facial paralysis	RCT	41/41	RYZBF + acyclovir vs. acyclovir	Higher total effective rate (97.56% vs. 82.93%, *P <* 0.05); Superior improvement in facial nerve function	R (2023)
	Female pudendal neuralgia	RCT	60/60	RYZBF + warm needle at baliao vs. warm needle at baliao	Clinical efficacy of combo group > acupuncture-only (*P <* 0.05)	Xiao and Tu (2025)
Other	Ischemic stroke (time-sequenced)	Clinical observational study	80 (40/40)	Tibetan med time-sequenced combo regimen vs. conventional	Improvement in total neurodeficit score more significant, higher total effective rate (97.5% vs. 80.0%)	Deng et al. (2022)

## 6 Safety evaluation

Acute toxicity tests showed that the maximum oral dose of RYZBF in mice reached 15 g crude drug/kg, equivalent to 181 times the clinical dose. No animal deaths or significant toxic reactions were observed at this dosage. According to the Guidelines for New Drug Research of Traditional Chinese Medicine, RYZBF belongs to the “practically non-toxic” category, indicating low acute toxicity and high clinical safety (Li et al., 2023).

In long-term toxicity tests, rats were continuously administered RYZBF orally for 180 days (25–100 times the clinical dose). The results showed no significant differences between the low, medium, and high dose groups and the control group in indicators such as general behavior, appearance, food intake, water consumption, and body weight gain. Blood biochemical tests revealed mild decreases in aspartate aminotransferase (AST), alanine aminotransferase (ALT), blood glucose, creatinine, and thrombin time (TT), and mild increases in blood chloride, fibrinogen (FIB), mean corpuscular hemoglobin concentration (MCHC), and kidney index in the dose groups. However, these changes were within the normal physiological range and returned to normal 30 days after drug withdrawal, with no other obvious chronic toxic reactions or delayed toxic effects observed (Duojieladan et al., 2014).

Clinical safety monitoring data indicate that the adverse reaction incidence rate for RYZBF is approximately 6.9%, primarily manifested as mild gastrointestinal discomfort or skin allergic reactions, with no reports of serious adverse events (Zhang et al., 2023). Compared to other anti-gout drugs, RYZBF has a lower incidence of adverse reactions in treating gouty arthritis and causes no liver or kidney damage (Li et al., 2021). Its application in special populations such as elderly patients also shows good safety (Chen et al., 2021). While these findings are encouraging, several limitations should be noted. The acute toxicity data are derived from animal experiments, and their extrapolation to humans requires confirmation. The long-term toxicity study, although conducted over 180 days, involved a relatively small sample size and lacked histopathological examination of major organs. Clinical safety data are primarily based on short-term observational studies, with no systematic evaluations in special populations such as pregnant women, children, or patients with hepatic or renal impairment. Furthermore, drug interaction studies are lacking, which is particularly relevant given the multi-component nature of RYZBF. Future research should include prospective safety studies in special populations and real-world data collection to further establish the long-term safety profile of RYZBF. It is worth noting that the following points require attention in clinical use: it is contraindicated in individuals allergic to any ingredient in the prescription; it should be used with caution in pregnant and breastfeeding women; consumption of raw, cold, spicy, and greasy foods should be avoided during medication; and potential drug interactions should be considered when used concurrently with other medications.

## 7 Discussion

### 7.1 Traditional Tibetan medicine theory and formula compatibility

As a treasure of the Tibetan medical theoretical system, the formulation philosophy of RYZBF reflects the principles of the Tibetan medical “holistic view” and “balance view.” Based on the Tibetan medical theories of “Taste, Property, Post-Digestive Taste” and property/efficacy compatibility, this formula combines thirty medicinal materials according to their properties and effects, forming a synergistic therapeutic system with heat-clearing, mind-awakening, collateral-dredging, and serous-fluid-drying properties. This compatibility theory—progressing from basic medicinal properties to holistic efficacy, and further to inherent efficacy—along with its structured, categorized compatibility framework, reflects the core concepts of Tibetan medicinal property compatibility and the therapeutic philosophy of balancing the “Three Nyépa (rLung, mKhris-pa, Bad-kan)”. It aims to treat diseases through holistic regulation involving multiple targets and pathways. Among the ingredients, *Margaritifera* and *L. brachystachya* focus on regulating the white channels and awakening the mind and opening the orifices, directly targeting the core pathogenesis of “White Channel Disorder.” Heat-clearing and blood-regulating medicines like *Moschus* and *C. sativus* synergistically exert detoxifying, tissue-regenerating, and chronic-heat-clearing effects. rLung disorder medicines like *S. aromaticum* and *M. fragrans* maintain white channel function by regulating the physiological state of rLung. Serous-fluid-drying medicines like Succinum and *Cassia obtusifolia* collectively achieve the effect of drying serous fluid and benefiting the joints (Zhuoma Cuo et al., 2023). This rigorous formulation structure not only reflects the systematic nature of Tibetan medical theory but also demonstrates its profound understanding of disease essence. Particularly, the Tibetan medical theory of time-sequenced medication organically combines human physiological rhythms with medicinal action characteristics. Studies show that using RYZBF in combination with classic formulas like Qishiwei Zhenzhu Pill and Ershiwei Chenxiang Pill according to a “morning-late morning-noon-evening” time sequence can significantly improve clinical efficacy (Gong et al., 2019; Guo et al., 2016). This medication mode is based on the Tibetan medical “Three Nyépa Chronology” theory, which posits that the human body has different physiological states and pathological characteristics at different times of the day. Precise timing of medication can maximize therapeutic effects. Modern chronobiology research is consistent with the view that the human body indeed possesses various biological rhythms, and drug metabolism and efficacy do differ significantly at different time points (Zhong et al., 2024). Therefore, Tibetan medicinal property compatibility, supplemented by theories such as Tibetan constitutional medicine and time-sequenced medication, not only has a profound traditional medical foundation but also highly aligns with modern research in chronopharmacology and precision diagnosis and treatment, providing important insights for optimizing clinical medication regimens.

### 7.2 Phytochemistry, pharmacological mechanisms, and safety

Modern research indicates that RYZBF contains various active metabolites such as flavonoids, alkaloids, terpenoids, and organic acids, with flavonoids and their glycosides constituting the largest proportion. Pharmacokinetic studies confirm that multiple metabolites can be effectively absorbed and distributed to brain tissue, providing a scientific basis for explaining its mechanism of action in treating central nervous system diseases. Regarding safety, toxicological evaluation indicates that the drug belongs to the “practically non-toxic” category. Clinical application shows a low incidence of adverse reactions, indicating good clinical safety. These studies provide important scientific support for the modern development and clinical application of RYZBF.

Modern pharmacological research has revealed the mechanism of action of RYZBF from multiple dimensions. In terms of neuroprotection, the drug not only inhibits neuronal apoptosis by regulating the expression of apoptosis-related proteins like Bcl-2/Bax but also promotes the expression of angiogenesis factors like CD34 and VEGF, improving cerebral microcirculation. Its anti-inflammatory action involves the fine regulation of inflammatory factor networks, inhibiting the release of pro-inflammatory factors while modulating the functional state of immune cells, forming a systematic anti-inflammatory immunomodulatory network. Furthermore, its effects on improving hemorheology and reducing oxidative stress damage collectively constitute its multi-mechanism, multi-target pharmacodynamic basis. These important findings not only validate the scientific nature of the Tibetan medical “Taste, Property, Post-Digestive Taste” compatibility theory but also provide new perspectives for understanding the pathogenesis of complex diseases. RYZBF and its combination regimens achieve holistic regulation of disease networks through the synergy of multiple metabolites, centrally embodying the “multi-component, multi-link, multi-target” action advantage of Tibetan medicine (Huang et al., 2024; Wu et al., 2019).

### 7.3 Clinical applications and evidence synthesis

Clinical studies have reported that RYZBF shows potential efficacy across multiple disease areas. For musculoskeletal system diseases, its therapeutic effects on osteoarthritis and gouty arthritis have been validated by multicenter clinical trials, effectively alleviating symptoms and improving laboratory indicators with good safety. In the treatment of cerebrovascular diseases, whether for acute-phase cerebral infarction or recovery-phase vascular dementia, RYZBF combined with conventional drug therapy shows synergistic effects, promoting neurological function recovery and improving patients’ quality of life. In the field of neurological disorders, the drug has demonstrated efficacy for various types of neuropathic pain, migraine, and peripheral neuropathy. Particularly, treatment regimens for White Channel Disorder based on Tibetan medical theory—focusing on harmonizing the Three Nyépa—show new application value in modern neurological disease treatment. These clinical outcomes reflect the Tibetan medical concept of “treating the root cause of disease,” achieving therapeutic effects through multi-target regulation and providing new approaches for treating complex diseases.

A critical limitation of this clinical evidence is the absence of Tibetan medical syndrome differentiation. All cited studies used only Western diagnoses and did not distinguish “heat-type” from “cold-type” White Channel Disorder. Since RYZBF is indicated primarily for heat-type cases (characterized by inflammation, redness, heat, and pain), this omission may have diluted effect sizes in mixed populations. Future trials should incorporate validated Tibetan diagnostic criteria for subgroup analyses to better define the formula’s therapeutic scope and improve evidence precision.

### 7.4 Clinical implications

The multi-target profile of RYZBF supports its use mainly as an adjunct to standard care in disorders that combine inflammatory, neurovascular, and pain components—notably osteoarthritis, post-stroke recovery, and neuropathic pain—where its favourable safety profile also favours longer-term use. As the available evidence is strongest for heat-type White Channel Disorder, clinicians applying Tibetan diagnosis should prioritise heat-type presentations and regard the formula as complementary to, rather than a substitute for, evidence-based conventional therapy until higher-level trials are available.

### 7.5 Limitations and future directions

Key limitations of current evidence at a glance.

- Phytochemical: Lack of non-targeted chemical fingerprinting; quantification limited to a few marker compounds (e.g., ellagic acid, piperine); pharmacodynamic relevance of brain-penetrating metabolites not empirically validated; variability in analytical methods across studies limits comparability.
- Pharmacological: Predominance of single-pathway investigations; absence of positive controls in many studies; narrow dose ranges; inadequate chemical characterization of test preparations; most studies use male animals only.
- Clinical: Many studies are small-sample, non-blinded, or observational; no cold/heat syndrome subgrouping (as discussed above); short follow-up periods; lack of drug-interaction data; time-sequenced regimens studied only in uncontrolled designs.
- Safety: No systematic safety evaluations in pregnant or breastfeeding women, children, or patients with hepatic/renal impairment; long-term safety data beyond 180 days are unavailable.
- Traditional theory integration: Direct experimental validation linking concepts such as “drying serous fluid” to specific molecular pathways (e.g., aquaporins, lymphatics) is currently missing.

Consistent with the ConPhyMP assessment, the phytochemical characterization of RYZBF remained incomplete across the reviewed studies, with none of the primary pharmacological studies reporting voucher specimens or chemical fingerprints.

Building on the limitations above, priority directions for future research are: (1) standardised phytochemical characterisation, including voucher specimens and full chemical fingerprinting, currently reported by none of the studies; (2) adequately powered, multicentre randomised controlled trials incorporating Tibetan syndrome differentiation (heat-versus cold-type) for prespecified subgroup analysis; (3) mechanistic studies conducted under pathological conditions, with blood–brain-barrier and target validation; (4) pharmacokinetic and quality-control standardisation across preparations; and (5) long-term safety evaluation in special populations.

To facilitate cross-disciplinary understanding, Table 5 below provides a conceptual mapping between key Tibetan medical concepts and their modern biomedical correlates, based on the evidence reviewed above.

**TABLE 5:** Conceptual mapping between Tibetan medical concepts and modern biomedical correlates for RYZBF.

Tibetan medical concept	Modern biomedical correlate	Evidence in RYZBF (section)
White channel	Central and peripheral nervous system	Neuroprotection, axon regeneration, remyelination, cognitive improvement (Neuroprotective and Repair Effects)
Drying serous fluid	Resolution of inflammatory exudation, joint swelling, edema, synovial effusion	Reduced joint swelling (OA model), decreased neutrophil aggregation, suppressed pro-inflammatory cytokines (Anti-inflammatory and Immunomodulatory Effects section)
Clearing heat (for heat-type white channel disorder)	Anti-inflammation, antipyresis, resolution of neuroinflammation, ischemic injury, inflammatory pain	Downregulation of NF-κB/TNF-α/IL-1β/COX-2, inhibition of microglial activation, modulation of CXCL10-CXCR3 pathway (Anti-inflammatory and Multi-mechanism Analgesic Effects)
Awakening the mind and opening the orifices	Neuroprotection, anti-apoptosis, promotion of neurotrophic factors	Upregulation of BDNF, NGF, VEGF; regulation of Bcl-2/Bax/caspase-3; improved neurological scores (Neuroprotective and Repair Effects)
Relaxing sinews and dredging collaterals	Analgesia, improvement of motor and sensory function	Multi-mechanism analgesia (NMDA, CXCL10, NGF/TrkA/TRPV1); improved Fugl-Meyer scores in stroke patients (Multi-mechanism Analgesic Effects and Clinical sections)
Harmonizing the three nyépa	Neuro-immune-endocrine network homeostasis	Multi-target, multi-pathway integrative regulation across inflammation, neural repair, and circulation (All pharmacological sections)

The mappings presented in this table are derived from Tibetan medical literature (e.g., the four tantras, contemporary Tibetan medical textbooks) and are cross-referenced with the pharmacological and clinical evidence cited in this review. The correspondences represent the authors’ interpretive synthesis based on the available data.

## 8 Conclusion

As a classic Tibetan medicinal formula for treating “White Channel Disorder,” The Ruyi Zhenbao Formula (RYZBF) exemplifies the convergence of centuries-old clinical application with modern scientific validation. This review comprehensively integrates Tibetan medicinal compatibility theory with ConPhyMP-guided evidence quality assessment for a multi-herbal formula. This review provides a comprehensive integration of its compatibility principles, phytochemical metabolites, pharmacological mechanisms, clinical evidence, and evidence quality assessment, establishing a comprehensive framework bridging traditional theory and modern science. The multi-level, synergistic, and progressive compatibility principles—from taste-based compatibility to efficacy-based compatibility—elucidate the formula’s multi-dimensional therapeutic characteristics and offer a modern scientific interpretation of the Tibetan medical concept of “holistic regulation.” Pharmacological studies have demonstrated that the multi-target mechanisms of RYZBF—neuroprotection, anti-inflammation, analgesia, and circulation improvement—closely correspond to core Tibetan medical concepts such as “awakening the mind,” “drying serous fluid,” and “relaxing sinews and dredging collaterals,” providing preliminary mechanistic insights relevant to White Channel Disorder. However, critical limitations remain in the current evidence base, particularly regarding phytochemical characterization and clinical study quality, underscoring the need for standardized, high-quality future research. The integrative framework established in this review may serve as a methodological reference for the modernization of other ethnic medicine formulations.
